# A three layered histone epigenetics in breast cancer metastasis

**DOI:** 10.1186/s13578-020-00415-1

**Published:** 2020-03-30

**Authors:** Debparna Nandy, Sruthy Manuraj Rajam, Debasree Dutta

**Affiliations:** Regenerative Biology Program, Rajiv Gandhi Centre for Biotechnology, Thycaud PO, Poojappura, Thiruvananthapuram, Kerala 695014 India

**Keywords:** Histone, Variants, Chaperone, Modification, Breast cancer, Metastasis

## Abstract

Thanks to the advancement in science and technology and a significant number of cancer research programs being carried out throughout the world, the prevention, prognosis and treatment of breast cancer are improving with a positive and steady pace. However, a stern thoughtful attention is required for the metastatic breast cancer cases—the deadliest of all types of breast cancer, with a character of relapse even when treated. In an effort to explore the less travelled avenues, we summarize here studies underlying the aspects of histone epigenetics in breast cancer metastasis. Authoritative reviews on breast cancer epigenetics are already available; however, there is an urgent need to focus on the epigenetics involved in metastatic character of this cancer. Here we put forward a comprehensive review on how different layers of histone epigenetics comprising of histone chaperones, histone variants and histone modifications interplay to create breast cancer metastasis landscape. Finally, we propose a hypothesis of integrating histone-epigenetic factors as biomarkers that encompass different breast cancer subtypes and hence could be exploited as a target of larger population.

## Background

Cancer has now become a worldwide phenomenon. According to World Health Organization, in the year 2018, 18.1 million new people have been found to be diagnosed with cancer globally [1]. Among women, breast cancer is considered to be the most common cancer and second most amongst overall [2]. The incidence rate of breast cancer is 1.7 million per year globally, which is a very huge number and need immediate attention [3]. With the advent of modern techniques, diagnosis, prognosis and treatment of breast cancer have considerably evolved till date. Upon early diagnosis, breast cancer gets cured but sometimes the disease relapses at some secondary sites by means of a phenomenon called “metastasis”, a Greek word meaning “displacement” [4]. Breast cancer metastasize to distant body parts including brain, lungs, bone and this makes it one of the deadliest disease that is hard to beat [5–8]. Various diagnostic options are available that has led to the betterment of the diseased condition. By far the most widely used option for the treatment of metastatic condition is chemotherapy and hormonal therapy [9]. In order to target the metastatic cells undergoing the cellular change in morphology called Epithelial to mesenchymal transition (EMT), one need to understand the molecular crosstalk leading to such cell fate [10]. A set of transcription factors play critical role in the process of EMT. They induce EMT through the transcriptional downregulation of E-cadherin (CDH1) while upregulation of mesenchymal-specific genes of Snail Family Transcriptional Repressor 1/2 (SNAI1/SNA2), Zinc Finger E-Box Binding Homeobox 1/2 (ZEB1/2), Twist basic helix-loop-helix transcription factor 1/2 (TWIST1/2), Forkhead Box C1/2 (FOXC1/2), Transcription factor 3 (TCF3), Goosecoid Homeobox (GSC) [11]. Additional to these transcription factors, a substantial number of recent studies provided evidence in the involvement of other types of proteins including proto-oncogene Cellular Oncogene Fos (c-FOS), Zinc finger protein 367 (ZNF367), ribosomal protein RPL15, RNA-binding protein A-Kinase Anchor Protein (AKAP8) in regulation of breast cancer metastasis [12–16]. However, for understanding the cellular transition mechanisms lying in the heart of breast cancer metastasis, it is very important to shed light over the epigenetic mechanisms besides these regulations. Currently, multiple studies have implicated the involvement of epigenetics in breast cancer metastasis. However, no such consolidated review is available from which one can summarize the entire histone epigenetics involved in the perspective of breast cancer metastasis. Therefore, this current review aims to generate a histone epigenetic landscape starting from its recruitment by histone chaperone, functional differences among histone variants to their modifications in the perspective of breast cancer metastasis.

Eukaryotic DNA is a tightly packaged, highly organized and regulated structure composed of DNA bound to histones and non-histones proteins in the form of nucleosomes [17, 18]. Nucleosome, the basic structural unit of DNA is formed when 147 bp of chromatin fiber, wound around a histone octamer composed of H2A–H2B dimer and tetramer of H3 and H4 histone molecules [19, 20]. Nucleosomes are connected to each other by means of linker histones H1 [21]. Nucleosomes along with linker DNA is termed as chromatosome, which is 166 bp in size [22]. Besides DNA compaction, nucleosome organization also helps in the recruitment of chromatin modifying enzymes by acting as a scaffold [23].

**Fig. 1:**
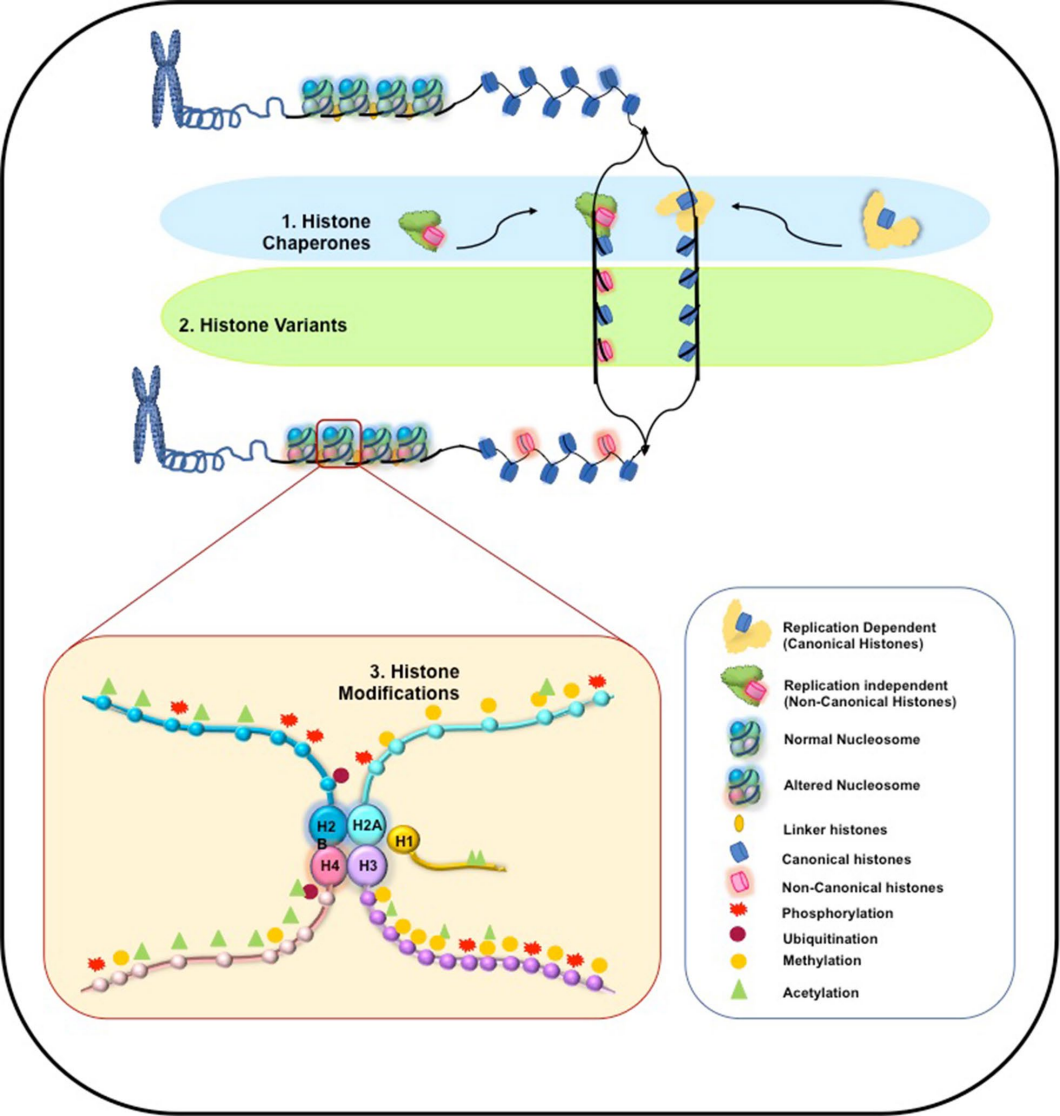
Layers of histone epigenetics composed of histone chaperones, histone variants and their modifications. (1) First layer of histone epigenetics is histone chaperone, which recruits histone variants into the chromatin. Canonical histones are recruited in replication dependent manner while non-canonical histone variants are recruited in replication independent manner. (2) Second layer of histone epigenetics is histone variants, which plays an important role of maintaining chromatin switch [ON/OFF state]. (3) Final layer of histone epigenetics is Histone Modifications which further modifies the chromatin state by addition of functional groups into the tails of histones

Histones are the key player responsible for the interaction with DNA and ultimately leading to the altered chromatin state of the cell [24]. Hence starting from the histone recruitment by histone chaperone to histone eviction, displacement and modification, altogether they play a great role in maintaining the chromatin state [open/closed] of a cell [25]. The beauty of epigenetics of histone is that, a variety of histone molecules along with their recruiters (histone chaperones) and modifications in different combinations comes to the scenario creating a unique pattern to determine the cell fate as depicted in Fig. 1. Disruption or modification of such epigenetics of histones has been found to be associated with many diseases including breast cancer [26].

### Epigenetics of histone

Histone epigenetics is a multi-layered regulatory process leading to the formation of open or closed chromatin state. It can be broadly divided into three layers (Fig. 1), with the first layer being histone chaperone (HC) [27]. HCs are defined as protein molecules responsible for histone metabolism. HCs play an important role in maintaining the chromatin dynamics of the cell by maintaining, transporting, recruiting and replacing the histones [28]. HCs can recruit the histone molecules at nucleosome independently or in association with other HCs [29]. Functionally histone chaperones have been implicated in replication, repair and in regulation of transcription as well [28].

Second layer of histone epigenetics comprises of the histones themselves (Fig. 1). Histones—the “key player”, are basic protein around which DNA is wounded form the stable nucleosome complex. Two distinct classes of histone molecules exist. The core histones called canonical histones whereas the regulatory histone variants (HVs) are termed as non-canonical histones. Canonical histone molecules are deposited during the S-phase of DNA replication [30]. They are the “wild type” of histones, which helps to maintain normal chromatin state. On the other hand, non-canonical histones are recruited/replaced throughout the cell cycle as depicted in Fig. 1 [30, 31]. So far implications of these histone variants have been widely reported in context of development and diseases [32].

In the final layer of epigenetics of histone, histone modifications (HMs) come into the picture (Fig. 1). Histone modification can be defined as the addition of chemical moiety to the protruding amino terminal tail of a histone molecule [19]. Various types of modifications have been reported so far. Histone modification include methylation, acetylation, phosphorylation, ADP ribosylation at particular amino acid residues [33–36]. These modification pattern leads to a change in the chromatin state on the basis of a particular “Histone Code”. “Histone Code Hypothesis” given by Allis and coworkers states that- “distinct pattern of covalent histone marks” affects the transcription efficiency of the gene leading to the change in transcriptomic profile of the cell [18]. It is quite interesting to see, how all these histone epigenetic players are interacting with each other in such a complicated manner (depicted in Fig. 1). However, often this class of epigenetic processes is either disrupted leading to a diseased condition or its disruption lead to a diseased condition. In short, epigenetic changes could be either the cause or the result of a diseased condition including breast cancer. In this review, we will discuss such epigenetic features of cells, which underwent disruption or modification in breast cancer metastasis condition.

### Histone chaperones in breast cancer metastasis

#### APLF

Aprataxin and PNK (Polynucleotide Kinase) like factor (APLF) is a histone chaperone [37]. Apart from histone chaperone activity, APLF is associated with the non-homologous end joining DNA repair pathway [33, 38]. The acidic C-terminal domain (AD) of APLF is responsible for histone chaperone activity [37]. APLF AD domain is found to be structurally homologous to NAP1 protein (Nucleosome Assembly protein 1), characterized as a histone chaperone [39].

**Fig. 2:**
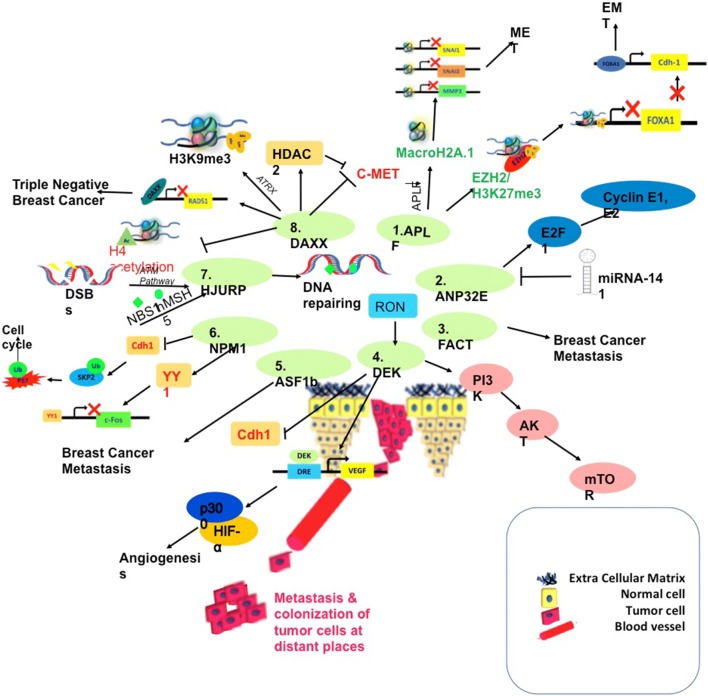
Histone chaperone landscape of breast cancer metastasis. (1) APLF downregulation causes recruitment of repressive histone MacroH2A.1 in the promoter of mesenchymal genes like *SNAI1, SNAI2* and *MMPs*, thereby promoting MET. Another pathway by APLF is showing, how repressive histone mark EZH2/H3K27me3 is recruited in the promoter region of FOXA1. In absence of FOXA1, CDH1 stops transcribing, thus pushing the cell towards EMT. (2) In ANP32E over-expressed cells, cell cycle regulator E2F1 causes upregulation of Cyclin E1/E2 therefore leading the cells to cell cycle. However, ANP32E itself is regulated at the transcript level by mi-RNA-141. (3) Over-expression of FACT has been associated with Breast Cancer Metastasis. (4) DEK has been found to directly inhibit CDH1, thus causing EMT. DEK also promotes angiogenesis by binding to DRE (DEK Response Element) in the VEGF promoter and recruiting p300 and HIF-α. Another pathway is showing how DEK induces metastasis via PI3K/AKT/mTOR pathway. (5) ASF1B is found to be upregulated in breast cancer metastasis. (6) NPM1 causes c-FOS suppression via YY1 expression. NPM1 also upregulates CDH1 levels, which causes SKP2 degradation, leading to p27KIP1 ubiquitination, thereby inducing breast cancer proliferation. (7) DNA Double Strand Breaks (DSBs) causes recruitment of ATM, which phosphorylates HJURP. HJURP along with DNA repairing protein hMSH5 and NBS1 repairs the DNA. (8) DAXXX inhibits c-MET [proto-oncogene] directly as well as via HDAC2 recruitment. DAXX binds to the promoter region of RAD-51 and forms repressive mark. However in breast cancer metastasis, downregulation of DAXX is found which causes over-expression of RAD-51 and the entire process corroborates the metastatic phenomenon

Although mass spectrometry study and immunoprecipitation analysis showed that APLF could interact with all the four histones (H2A, H2B, H3 and H4) but it could only recruit H2A–H2B histone dimer with higher affinity [37]. The Cancer Genome Atlas (TCGA) database analysis for invasive ductal breast cancer patient sample comprising of a cohort of 815 patient samples, showed significant increase of APLF in triple negative breast cancer (TNBC) sample and invasive breast cancer of the basal type [40]. The breast cancer molecular subtypes include triple negative/basal, luminal A, luminal B and HER2-enriched. Luminal A tumor can be either Estrogen-receptor positive (ER^+^) or HER2 negative (HER2^−^). Luminal B tumors could be ER^+^ or HER2^+^ or HER2^−^. Triple negative breast cancers are ER^−^, HER2^−^ and progesterone receptor negative (PR^−^). Human breast cancer tissue sections reflected consistently enhanced expression of APLF in comparison to their adjacent control sections [40]. APLF expression was highest in TNBC cell line MDAMB-231 with respect to invasive cell line MCF-7 and normal mammary epithelial cell line MCF-10A [40]. In short, APLF expression was directly proportional to the degree of metastatic nature of the breast cancer cells [40]. APLF regulated the genes implicated in epithelial-to-mesenchymal transition (EMT) associated with breast cancer metastasis. Enhanced recruitment of repressive HV MACROH2A.1 (encoded by *H2AFY* gene) at the mesenchymal specific gene promoters including *SNAI1, SNAI2, MMP3* (Matrix metalloprotease 3) and *MMP9* (Matrix metalloprotease 9) abrogated the expression of these genes involved in metastasis [40]. On the other hand the epithelial marker, CDH1 was induced upon loss in APLF expression due to the binding of master regulator forkhead box protein A1 (FOXA1) within the *CDH1* locus in MDAMB-231 cells [40]. Basically, loss in APLF resulted in the derepression of the *FOX1* promoter due to the erasure of H3K27me3 mark aided by the loss in expression of EZH2 (Enhancer of Zeste Homolog 2), the histone methyl transferase of the Polycomb Repressor Complex 2 [40]. This case of epigenetic regulation of breast cancer metastasis is a classical example as it distinctively shows how all three layers of histone epigenetics work in connection to one another HC to HV to HM (Fig. 2).

#### HJURP

Holliday junction recognition protein (HJURP) is a histone chaperone that widely functions in the eviction, recruitment and replacement of histone molecules [40, 41]. HJURP is known for the deposition of Centromere protein A (CENP-A) histone variant in the centromeric nucleosomes [41]. CENP-A binding domain (CBD) is a stretch of 80 amino acid region located at the N-terminal of HJURP protein responsible for recruiting CENP-A [41]. Aberrant HJURP expression has been observed in a cohort of 71 breast cancer patients. Increased HJURP expression in luminal A subtype breast cancer progression associated with the increase in probability of metastasis. In fact, HJURP has been reported to be a better biomarker than Ki67 for assessing the proliferation rate in luminal A breast cancer [42, 43].

Higher expression of HJURP has been associated with breast cancer progression of different subtypes (ER^−^, PR^−^) and associated with shorter survival [43–45]. Bravaccinni et al., shown that patients expressing higher HJURP in the stroma stands more than sevenfold higher risk in chances of a breast cancer relapse [45]. Both in vitro and in vivo data has shown that higher HJURP level is more sensitive to radiation therapy [40]. Like APLF, HJURP is also found to have DNA repairing function. Upon induction of DNA double strand break, Ataxia Telangiectasia Mutated (ATM) get recruited to the site which phosphorylates its downstream targets including HJURP [40]. HJURP along with its interaction partners MutS Homolog 5 (hMSH5) and Nibrin (NBS1), a part of MRN complex, starts the process of homologous recombination. hMSH5 along with hMSH4 is known to recognize Holliday Junction Complex (Fig. 2) [46]. HJURP activation is a major event for maintaining chromosomal stability and thus support its induced expression in cancer cells undergoing frequent genomic instability [47].

#### DAXX

Death domain-associated protein (DAXX) is a histone chaperone that recruits H3.3 histone variant [48]. DAXX interacts with Alpha Thalassemia/mental Retardation syndrome X-linked (ATRX), a chromatin remodeler [48–50]. Among the four domains of DAXX protein, the histone-binding domain HBD binds to the H3.3/H4 histones dimer. DAXX have two Sumo Interacting Motifs (SIM) at either terminal important for recruiting DAXX into heterochromatin segment of the chromosome [49]. The acidic C-terminal domain of DAXX interacts with p53 and H3.3/H4 tetramer [49].

Unlike other histone chaperones DAXX is found to be downregulated in breast cancer metastasis [51]. DAXX act as a negative regulator of MET proto-oncogene (c-MET). DAXX causes c-MET repression by interacting with the proximal promoter region of *c*-*MET* [51]. DAXX interact with the histone modifying enzyme, Histone deacetylase 2 (HDAC2) thereby recruiting HDAC2 at the *c*-*MET* promoter resulting in repression of the locus due to loss in H4 acetylation [51]. A similar trend was observed in metastatic breast cancer tissue samples [51].

DAXX is found to function as a tumor-suppressor [52]. Upon over-expression of DAXX in TNBC cells, MDAMB-231 and MDAMB-157, significant reduction in cell growth, colony formation and tumor formation was observed [52]. Mechanistically, DAXX bind to the *RAD51* promoter thereby repressing the function of RAD51 and resulting in the inhibition of breast cancer metastasis (Fig. 2) [52, 53].

#### DEK

DEK proto-oncogene (DEK) protein is another H3 histone chaperones [54]. It specifically binds to H3.3 variant by means of four-way cruciform structure [55, 56]. This cruciform structure mainly helps in forming positive supercoiling of DNA, thus shifting the chromatin towards closed state. In fly, DEK function as suppressor of variegation thereby maintaining the heterochromatin integrity of the genome [57]. DEK interact with HP1α (Heterochromatin protein 1α) that augments the interaction of the complex with H3K9me3 [57]. H3K9me3 being a repressive chromatin mark justifies the suppressive role of DEK. Additionally, DEK interacts with HDAC2 and is responsible for H3–H4 specific acetyltransferase inhibitor activity [58]. Both of these factors further intensify the repressed state of the chromatin. Interestingly, DEK is also involved in transcriptional repression by interacting with DAXX [58].

DEK has been implicated in various kinds of cancer including breast cancer metastasis [58]. DEK is significantly enhanced in TNBC MDA-MB-231 cells. DEK regulated cellular invasion has been partially attributed to β-Catenin activation in the cancer cells [59]. RON (Receptor d’ Origin nantaise), a Tyrosine kinase receptor has been implicated in tumour progression and is a target of DEK [60]. In MMTV (malignant breast cancer mouse model) Ron mouse, upregulated DEK expression resulted in the induced probability of developing distant metastasis in comparison to RON DEK^−/−^ mice [60].

Positive correlation between DEK, breast cancer and lymph node metastasis have been reported by multiple groups [60–64]. DEK regulate EMT associated with breast cancer metastasis via PI3K/AKT/mTOR pathway in TNBC cells (Fig. 2) [61]. Additionally, DEK regulate tumor angiogenesis in breast cancer by binding to the DEK Response Element (DRE) in the *VEGF* promoter thereby inducing HIF-α and acetyltransferase P300 recruitment within the promoter (Fig. 2) [62]. A positive correlation with angiogenesis factor VEGF and formation of micro-vessel among a cohort of 58 breast cancer patients further validated the role of DEK in breast cancer [62, 65]. This induced expression of DEK positively correlate to poor prognosis in TNBC patients as well in invasive ductal carcinoma patients [62, 65]. Additionally, invasive adenocarcinoma patient also exhibited higher expression of DEK [59].

#### ANP32E

Acidic leucine rich Nuclear Phosphoprotein 32 (ANP32E) belongs to ANP32 family (member E) [66, 67]. ANP32E is H2A.Z-H2B specific histone chaperone binding through the C-terminal domain. The region of C-terminal domain interacting with H2A.Z is known as ZID (H2A.Z Interacting Domain). H2A.Z has an αC-helix region inside M6 cassette region (89–100 aa) which specifically binds to ANP32E [67]. Once αC-helix region has interacted with H2A–H2B dimer, there is no provision left for the interaction with H3–H4 [67]. ChIP-sequencing results have shown ANP32E modulates H2A.Z recruitment at promoter, enhancer and insulator sites [67]. Cells when irradiated with UV radiation, H2A.Z is displaced by ANP32E followed by deposition of H2A.X by FACT [68]. Basal type breast cancer patient samples and TNBC cell lines including MDAMB-231, MDAMB-468, express high level of ANP23E [69]. Interestingly, ANP32E expression, could demarcate a clear distinction between TNBC and non-TNBC patient samples [69].

Interestingly, ANP32E is a part of Landmaine’s “six gene signature” that is used for the screening of distant relapse of breast cancer metastasis [70, 71]. ANP32E along with desmocollin 2 (DSC2), UDP glycosyltransferase 8 (UGT8), Integrin subunit beta 8 (ITGB8) and Fermitin family member 1 (FERMT1) is found to predict lung metastasis of breast cancer patients and has been used as prognostic marker [71]. ANP32 enhanced proliferation of TNBC cells by promoting G1/S transition through transcriptional induction of E2F1 and thereby resulted in induced tumorigenesis of TNBC cells [69, 72, 73].

#### ASF1

Another histone chaperone that has been implicated in breast cancer metastasis is Anti Silencing Factor 1 (ASF1) [74]. Mammalian ASF1 has two isoforms namely ASF1A and ASF1B in contrast to its yeast counter part which has only one [75]. ASF1 is known for recruiting H3–H4 histones into the nucleosome. ASF1 interacts with H3–H4 in heterodimeric manner instead of highly stable H3–H4 heterotetramer observed in normal nucleosomal packaging [74]. It performs multitude of functions like gene silencing, DNA replication and repair. NMR studies have shown the interaction between conserved core (amino acid 1–156) of ASF1A along with the C-terminal helix of H3 [74]. Other histone chaperones for H3–H4 tetramer are HIRA (Replication independent) and CAF1 (Replication dependent). ASF1A–HIRA complex function in a replication independent manner, whereas ASF1B-CAF1 in a replication dependent manner [75]. ASF1A influence cell recovery from DNA damage while ASF1B is associated with defective cell growth, sensitivity during replication stress and breast cancer metastasis [75, 76]. ASF1B level is significantly enhanced in tumor samples and metastatic breast cancer cell lines significantly increase the likelihood of developing breast cancer metastasis [75].

#### FACT

Facilitates chromatin transcription (FACT) protein, is a histone chaperone which acts as both nucleosome assembly and disassembly factor [77]. FACT has role in transcription elongation as well as DNA repair [78]. Upon DNA damage, FACT activates tumour suppressor protein, P53 and starts recruiting histone variant H2AX–H2B [78]. FACT is a heterodimeric complex of two other proteins—Suppressor of Ty16 homologue (SPT16) and structure specific recognition protein 1 (SSRP1) [78]. It binds to H2A–H2B dimer and H3–H4 tetramer with similar affinity. Both these protein binds at different location in the FACT molecule. FACT is reported to be upregulated in breast cancer patients [79]. Expression of SSRP1 subunit of FACT showed a strong correlation between matched primary and metastatic lesions in a large cohort of patients (n = 1092) [79]. Immunohistochemical studies showed SSRP1 is a reliable indicator of FACT level in tumors and not SPT16.

#### Nucleophosmin

Nucleophosmin (NPM1) is a nuclear phosphoprotein that shuttles between nucleoli and nucleoplasm [80, 81]. It is also a histone chaperone that is found to be interacting with histones H3, H2B and H4 [81]. Acetylation of Nucleophosmin by p300 changes the chromatin into open state thereby enhancing transcription process [81]. NPM1 has also been implicated in breast cancer metastasis [82–84]. Higher NPM1 expression has been found in the serum samples of breast cancer patients [80]. Study done among 100 breast cancer patient serum samples has shown association of Nucleophosmin auto-antibodies with the disease recurrence [81]. In breast cancer MCF-7 cell line, Nucleophosmin level is induced by estrogen and repressed by anti-estrogen [80, 85].

IHC data has shown higher NPM1 expression among TNBC patients [82]. In fact, NPM1 expression has been found to be higher among basal breast cancer patients than luminal [82]. Real time expression from tissue samples has shown higher *NPM1* expression in breast cancer (n = 1097) samples than normal (n = 114) [82]. NPM1 expression shares a positive association with proliferation index and ki-67 expression. Mechanistically, loss in NPM1 induced CDH1 expression that could accelerate SKP2 degradation causing p27^kip1^ubiquitination and finally resulting in induced cell proliferation [82]. NPM1 is also found to regulate YY1 transcription factor expression, which causes suppression of c-FOS expression thereby promoting cell growth [84]. However, Karhemo et al., has postulated NPM1 as a tumour-suppressor gene [85]. Lower NPM1 level associate with poor prognosis as studied among a cohort of 1160 breast cancer patient samples [85].

From the expression level of these histone chaperones in breast cancer metastasis we could infer that different factors including histone variants, histone modifications, histone modifying enzymes and signaling pathway interact in order to create a metastatic niche. We represent a landscape to capitulate how all the histone chaperones are responsible breast cancer metastasis (Fig. 2). All these histone chaperones along with their mechanism of inducing breast cancer and breast cancer associated metastasis has been stated in Table 1.

**Table 1:** Histone chaperones in breast cancer metastasis

S. no	Histone chaperone	Role in breast cancer metastasis	References
1.	APLF	APLF over-expression is associated with breast cancer metastasis Regulate MacroH2A.1 recruitment in EMT specific promoter, EZH2/H3K27me3 level at *FOXA1* promoter and recruitment of FOXA1 at CDH1 promoter	[40]
2.	HJURP	Higher expression in breast cancer metastasis condition Target of ATM signaling pathway, where it interacts with hMSH5 and NBS1	[43, 44, 46, 47]
3.	DAXX	Down-regulated in breast cancer metastasis Forms complex with ATRX in order to maintain H3K9me3 methylation Negative regulator of c-met DAXX knockout cells have lower H4 acetylation and higher HDAC2 activity Binds at the promoter region of RAD51	[50, 52, 53]
4.	DEK	Act as proto-oncogene Higher expression in breast cancer metastasis condition DEK knock down cells has lesser H3K9me3 mark, lower CDH1 expression. Induces metastasis via β-Catenin pathway Downstream target of RON Also found to mediate EMT via PI3K/AKT/mTOR pathway Causes angiogenesis via VEGF pathway	[55, 58, 60, 61, 63]
5.	ANP32E	Positive correlation with breast cancer metastasis Helps in the removal of H2AZ variants so that FACT can deposit ɣH2AX in response to DDR Influences E2F1 transcription factor Regulation by mi-RNA-141 Part of “Landmaine’s six gene signature” for predicting lung metastasis of breast cancer	[69, 71, 73]
6.	ASF1	ASF1B over-expressed in breast cancer metastasis	[75]
7.	FACT	Upregulated in breast cancer metastasis patient samples	[79]
8.	NPM1	Higher NPM1 expression among TNBC patients NPM1 expression more in basal breast cancer than luminal breast cancer samples NPM-1 regulates YY1 expression which causes suppression of c-FOS expression, hence promoting cell growth	[80, 82–84, 86]

### Histone variants implicated in breast cancer metastasis

#### H2A family

##### I. H2A.Z

H2A.Z histone variant was for the first time reported by West and Bonner, 1980 [86]. It is encoded by two genes *H2A.Z1* and *H2A.Z2* [87]. Although H2A.Z accounts for only 5% of the total H2A canonical histones, it is expressed throughout the entire cell cycle [88]. H2A.Z sequence is highly similar H2A histone sequences. Large number of studies carried on H2A.Z delineated how beautifully our genome is regulated in different conditions. H2A.Z histone enrichment has been found in the promoter, enhancer as well as insulator region [89]. H2A.Z is also found to be associated with pericentric heterochromatin domain [89]. So far acetylation of H2A.Z has been shown to have role in the maintenance of chromatin states wherein hypoacetylated H2A.Z accumulates in the heterochromatin region [89].

**Fig. 3:**
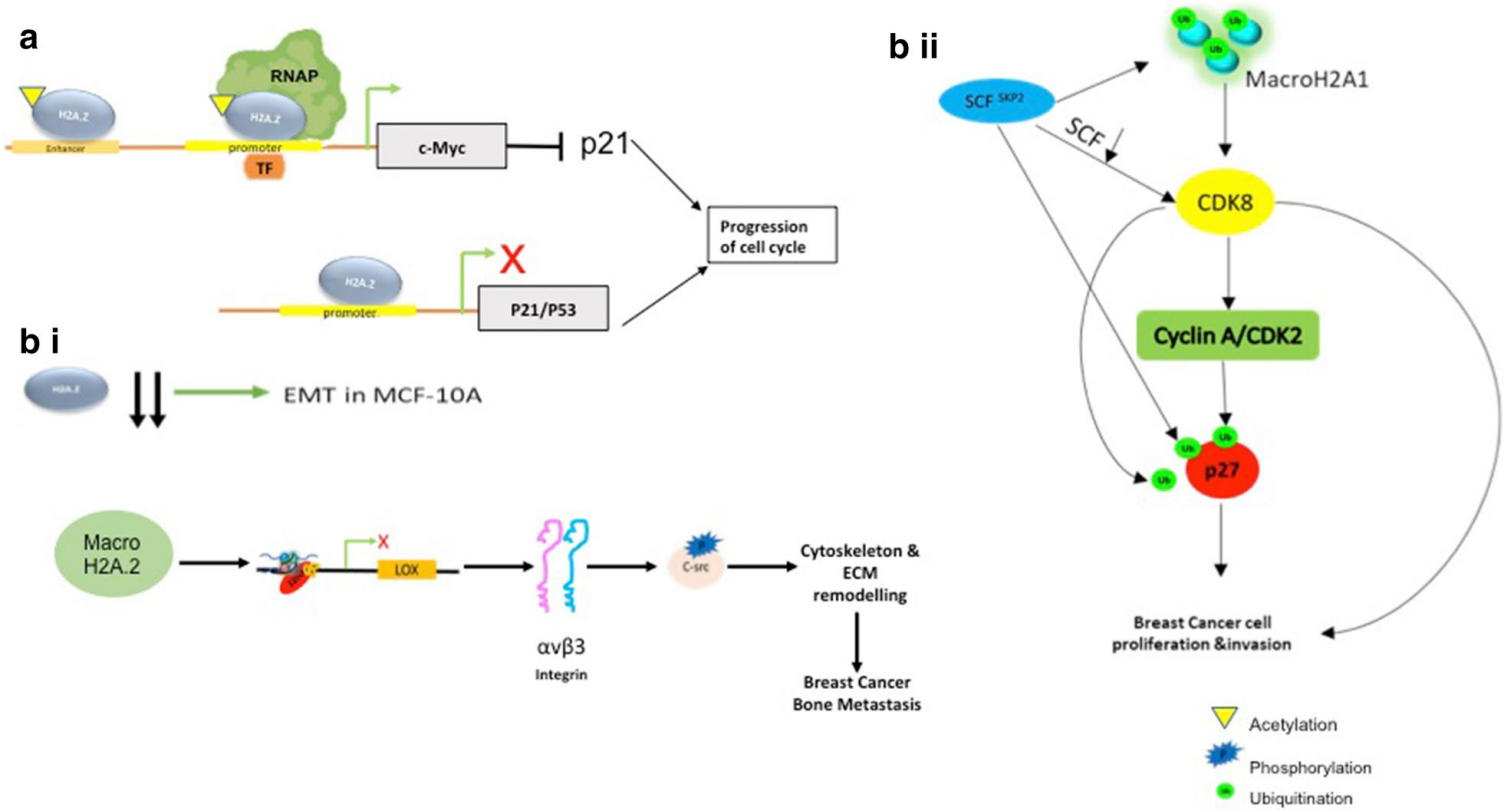
Histone variants in the regulation of cell cycle in breast cancer. **a** Enrichment of H2A.Z in the *c*-*MYC*promoter induces c-Myc expression. c-MYC in turn inhibits p21 [cell cycle inhibitor] thereby leading the cells into cell cycle. H2A.Z also causes p21/p51 transcriptional repression by binding to the later’s promoter region. H2A.Z knockdown induce EMT in MCF-10A. **b** (i) MacroH2A.2 recruits EZH2 which causes H3K27me3 repressive mark in the promoter region of *LOX* gene. LOX causes change in the αvβ_3_ integrin which causes phosphorylation of c-SRC causing cytoskeleton and ECM remodeling and finally leading to breast cancer bone metastasis. **b** (ii) SCF SKP2 causes ubiquitination and degradation of MacroH2A1, leading to CDK8 upregulation followed by Cyclin A/CDK2 recruitment, which causes cell proliferation. Besides, SCF SKP2, CDK8 and Cyclin A/CDK2 also ubiquitinates cell cycle inhibitor p27

Enhanced expression of H2A.Z is prevalent in breast cancer patient samples, in cell lines as well as in lymph node metastasis [90, 91]. H2A.Z is enriched at the *P53/P21* promoter thereby causing transcriptional repression [92]. Depletion of p21 results in cell cycle re-entry (depicted in Fig. 3a). Thus, high H2A.Z is found to be associated with cell proliferation [87]. Another way, by which H2A.Z regulate cell cycle is via activation of oncoprotein C-MYC expression [93, 94]. H2A.Z is found to activate oncoprotein *c*-*MYC* promoter. c-MYC in turn suppresses P21 thereby causing the cell to enter cell-cycle (Fig. 3a) [94]. H2A.Z is also found to be deposited within the promoter of *TFF1* (Trefoil factor 1), an ER-α positive breast cancer tumour marker [94]. Histone methyltransferase SMYD3 methylates H2A.Z.1 at Lys101 leading to a stable H2A.Z-chromatin association and thereby driving the cells to enter s-phase of cell cycle [90]. Positive enhanced correlation between H2A.ZK101me2 with SMYD3 has been observed in human breast tissue biopsies [90]. However contradicting role of H2A.Z has also been reported recently by Domaschenz et al. 2017 [87]. They confirmed that H2A.Z depletion imitates the EMT condition that is being produced by TGF-β pathway induction whereas overexpression induce the epithelial genes [87].This study could be further supported by the fact that loss in H2A.Z in MCF-10A cell line, EMT was induced (Fig. 3a). So, it is very much evident that H2A.Z indeed play an important role in chromatin maintenance and oncogenesis induction. Nevertheless, the precise role of H2A.Z is complex and need further intensive study. In order to get a clear picture, it is very important to have knowledge about the upstream regulator and downstream effector of H2A.Z. Breast cancer is a multifactorial disease, thus it is very important to have proper stratification of the sample before going for any kind of case–control analysis.

##### II. MacroH2A

MacroH2A is another H2A variant that has been implicated in breast cancer metastasis [95]. It has two variants namely MacroH2A1 and MacroH2A2 encoded by H2AFY and H2AFY2 respectively [96]. MacroH2A1 has again two splice variants MacroH2A1.1 and MacroH2A1.2 [96, 97]. The only difference between both of these splice variant is that MacroH2A1.2 lacks the Leucine zipper motif present in macroH2A1.1 [98]. In contrary to other histone molecules, MacroH2A has large non-histone parts [99]. Macro-histone molecules are also heavier (42KDa) than normal histone variant (15KDa) [99]. MacroH2A plays important part in nuclear organization and chromatin maintenance [99]. It is mainly associated with repressive chromatin mark, thus accounting for its presence in the heterochromatin region like inactivated X-chromosome [98]. Multiple studies have reported the tumour suppressor role of MacroH2A in breast cancer [95]. MacroH2A1 expression is inversely related to aggressiveness of breast cancer cell lines [95, 96]. Splice variant MacroH2A1.2 recruits EZH2 which trimethylates H3K27, leading to the formation of repressive chromatin mark around *LOX* (lysyl oxide) transcription start site [96]. LOX has been considered to be a major factor for breast cancer bone metastasis. In cancer cells, depletion of MacroH2A1.2 leads to LOX upregulation that inhibit metastasis via c-Src signaling pathway (Fig. 3bi) [96]. MacroH2A interaction with Her2 interaction leads to the activation of ERBB2, implicated in breast cancer metastasis [100, 101]. SKP2, a F-box protein known for degrading its different substrates including macroH2A1, is the upstream regulator of macroH2A1 [102]. SKP2 knockdown causes breast tumour suppression by upregulating macroH2A1 [102]. Additionally, SKP2 depletion leads to CDK8 upregulation, which helps in the ubiquitination of cell cycle inhibitor P27 (Fig. 3bii). Dardenne et al. 2012 has very beautifully illustrated how alternative splicing of macroH2A1 has important role in tumor invasiveness [103]. Decreased MacroH2A1.1:MacroH2A1.2 ratio has been associated with metastatic condition. However, contradicting reports indicated that MacroH2A1.1 level is positively correlated to TNBC patient samples [104]. Upregulation of MacroH2A1.1 is found to be associated with down-regulation of epithelial marker, E-cadherin and up-regulation of mesenchymal marker like *TWIST1* and *SNAI1* [104].

##### III. H2A.X

H2A.X has been widely implicated in multiple types of cancer [36, 105, 106]. ɣH2A.X is reported to be found at the site of double strand breakage, chromosomal ends and depleted telomeric region [107]. Phosphorylation of H2AX at Ser 139 (ɣH2A.X) by ATM leads to the formation of ɣH2A.X molecule which forms foci like structure [108]. ɣH2A.X serve as an signal that aids in the recruitment of DNA repairing machinery to the site of breakage [109]. Higher ɣH2A.X level has been reported in breast cancer patient samples and in triple negative breast cancer cells as well [110, 111]. ɣH2A.X is also been reported to function via TRAF6-ATM-H2AX signaling axis mediated by Hypoxia inducible factor 1α (HIF1α) [108]. HIF1α has been implicated in breast tumorigenesis and metastasis having a significant positive correlation with H2AX [108]. In fact, HIF1α substrates are deregulated in H2A^−/−^ mouse. TRAF6-a HIF1α target is self-ubiquitinated and gets activated under hypoxic condition. Activated TNF receptor associated factor 6 (TRAF6) then mediates mono-ubiquitination of H2A molecule, which in turn recruits ATM to H2AX and thus results in ɣH2A.X formation. This molecular interplay within different signaling molecule, transcription factor and histone variant leads to oncogenesis and metastasis.

#### H2B family

Very few literatures are available on the role of H2B histone variants in breast cancer metastasis [112]. Hypomethylation and upregulation of histone H2B variant, HIST1H2BJ is found to be associated with brain metastasis of breast cancer [112]. Wu et al. 2015 also confirmed the enhanced expression of histone H2B in breast cancer patient samples [113]. Interestingly, one study has delineated the role of another histone H2B variant, HIST1H2BE in hormone resistant cell lines [114]. HIST1H2BE is found to be hypomethylated and induced in cell-lines C4–12 (ER-α negative MCF-7) and LTED (long-term estrogen-deprived MCF-7 cells) compared to control MCF-7 cells [114]. Upon HIST1H2BE knockdown and overexpression in LTED and MCF-7 cell lines respectively, opposite trends in cell proliferation was observed. So, in effect histone H2B variants showed effect on metastasis but the mechanism is yet to be revealed as per our knowledge.

#### H4 family

A very recent study has shown the evidence of a new H4 histone variant-H4G [115]. Structurally, H4G does not have the C-terminal region of the normal H4 histone. Induced H4G expression is associated with breast cancer cell lines like MCF-7, LCC1 (MCF-7 ER−ve) and LCC2 (MCF-7 ER−ve and tamoxifen selected) when compared to normal MCF-10A cells [115]. Breast cancer patient samples also showed higher expression of H4G with respect to normal tissue samples. Upon H4G knock-out in MCF-7 cell line, cell proliferation was significantly reduced. Histone chaperone Nucleophosmin1 is found to be interacting with the α-helical-3 domain of H4G leading to its recruitment at the chromatin [115]. Although H4G has been found to be associated with breast cancer but so far no evidence has shown up regarding its metastatic potential.

#### H3 family

##### CENP-A

Has been implicated in breast cancer metastasis [116]. CENP-A is a H3 specific histone variant that is found in the centromere region [116]. CENP-A is induced in breast cancer tissue samples. CENP-A is also part of commercially available kit for the detection of early stage breast cancer [116]. Higher CENP-A is observed in ER^−^ tumor with respect to ER^+^ ones [117]. CENP-A showed positive correlation to ki-67 expression and corresponded to poor patient outcome [117].

The newly established histone variant H1° is a linker histone variant [118]. Breast cancer cells demonstrated significantly higher H1° expression but no further understanding to metastasis or its associated mechanisms have been revealed yet [118].

A list of all the histone variants has been summarized in Table 2.

**Table 2:** Histone variants in breast cancer metastasis

S. no.	Histone variants	Role in breast cancer metastasis	References
1.	H2A.Z	Over-expressed in breast cancer metastasis H2A.Z enrichment is found at p53/p21 and TFF1 gene promoters Regulate cell cycle via c-myc H2A.Z depletion imitates EMT	[87, 90–94]
2.	MacroH2A	Act as tumour suppressor Recruits EZH2 which causes H3K27 tri-methylation around LOX transcription start site HER2 interaction with MacroH2A causes activation of ERBB2 SKP-SCF complex is found to be an upstream regulator of MacroH2A and CDK8 as downstream effector mH2A.1 is found to be associated with the upregulation of EMT specific markers- Twist1 and Snail and downregulation of mesenchymal markers like E-cadherin	[40, 95, 96, 101, 102]
3.	H2A.X	Found at the site of double Strand breakage, telomeric erosion Forms ɣH2A.X upon phosphorylation by ATM at SER139 Higher ɣH2A.X level is associated with Breast Cancer as well as TNBC Reported to function via TRAF6-ATM–H2AX pathway mediated by HIF1α	[107, 108, 110, 111]
4.	H2B	Hypomethylation and upregulation of HIST1H2BJ is found in brain metastasis of breast cancer Hypomethylated and upregulated HIST1H2BE is found to be associated with breast cancer cell lines	[112, 114]
5.	CENP-A	Over-expression in breast cancer tissue samples Higher CENP-A expression is found in estrogen negative breast cancer condition than estrogen positive condition Positive correlation of CENP-A has been observed with ki-67 expression	[116, 117]
6.	H4G	Over-expressed in breast cancer cell lines	[115]
7.	H1	Over-expressed in cancer cells	[118]

### Histone modifications and their role is breast cancer metastasis

#### A. Methylation

Methylation is a type of post translational modification (PTM), which can alter the chromatin architecture of the cell [119]. There are two types of methylation observed, DNA methylation and histone methylation. Here in this review, we will restrict our discussion on histone methylation only. Methylation status of a cell is identified by the outcome of balance between methyltransferases (“writer”) and demethylases (“eraser”) [120]. Finally these signals are identified by “reader” molecule i.e. chromatin remodeler [120]. Histone methylation is found among basic amino acid lysine, arginine and histidine [121]. Numerous studies have demonstrated the significant role of histone methylation in breast cancer progression and metastasis [122, 123].

Spangle et al. [124] has described how PI3K/AKT signaling plays role in breast cancer progressionvia increase in H3K4me3 level. Enhanced H3K4me3level is found to be associated with breast tumors [124]. H3K4me3 is predominantly present in the gene promoters including *AURKB* and *E2F2*, an AKT target as well as has role in cell proliferation [124]. AKT i phosphorylate KDM5A, an H3K4 demethylase. Phosphorylated KDM5A is retained in the cytoplasm and does not enter in the nucleus thereby resulting in increased H3K4me3 level in the nucleus [124]. However, in presence of AKT inhibitor KDM5A translocate to nucleus and demethylates H3K4. Histone demethylase JARID1A/B are oncogenes, highly expressed in breast cancer condition [125]. They interact with pRB by demethylation of H3K4 [125]. This results in repression of E2F1 target genes by pRB protein.

Various studies have associated lower H3K27me3 expression with breast cancer progression, big tumour size, positive lymph nodes and estrogen negative tumours [126, 127]. H3K27me3 is a repressive mark catalyzed by EZH2 methyltransferase. EZH2 itself has been found to be upregulated in breast cancer and promote EMT [128, 129]. Apart from these, enrichment of H3K27 tri-methylation mark within the promoter of *FOXC1, RAD51, CDH1 and RUNX3* lead to increase in cell proliferation and metastasis [130].

Histone methylase Disruptor silencing 1 like (DOT1L) has been implicated in breast cancer and lymph node metastasis [131]. It donates methyl group to histone H3K79 from SAM. DOT1L interacts with c-MYC and p-300 for activating EMT in breast cancer [132]. In fact, in normal MCF-10A cells, overexpression of DOT1L could induce EMT [132]. Higher H3K79 di-methylation is also observed in the promoter region of BCAT1 gene [133]. BCAT1 being a c-MYC target has already been implicated in breast cancer [125]. H3K79me2 induce genomic instability and promote tumorigenesis [134].

Lower H4R3me3, H4R3me2 and H3K4me2 expressions associate with poor prognosis of breast cancer tumour [135]. IHC staining of H4R3me2 has shown lower expression in basal carcinomas and HER-2 positive tumours [135]. While higher expression of H4R3me2 is associated with lymph node stage [135].

Wang et al. [136] in their recent review has shown the roles of existing PRMT signaling in breast cancer via different histone modifications. PRMT1 as such is implicated for inducing tumorigenesis. Recruitment of PRMT1 within the *ZEB1* promoter incorporates H4R3 dimethylation mark that could drive EMT. PRMT5 on the other hand is found to induce breast cancer metastasis in TGF-β dependent manner [136]. PRMT5 is recruited to the *FOXP1* promoter where it causes H3R2 dimethylation and H3K4 tri-methylation and as a consequence leading to an accelerated cell proliferation [136]. Non-canonical histone variant H2A.Z undergoes methylation by SMYD3 to form H2A.Z.1.K101me2 in the promoter region of *CYCLIN A1*. As a result CYCLIN A1 binds to CDK1 and CDK2 gets activated and the cell enters cell cycle (G2/M phase and S-phase) [90].

#### B. Acetylation

Histone acetylation is also a PTM that is found to have wide role in transcriptional regulation of gene [137, 138]. Acetylation is the outcome of balance between writer histone acetyl transferase (HAT) and eraser histone deacetylase (HDAC) molecule [139]. HAT activity is associated with open chromatin structure owing to its donation of acetyl group to the histone tail while HDAC reverses the action of HAT activity and leads to the closed conformation of the chromatin structure. Histone acetylation has double role in the induction of carcinogenesis [140]. Acetylation can mediate both repression of tumour suppressor and activation of proto-oncogene [137, 141].

The non-metastatic MCF-7 cell lines acquire EMT like phenomenon due to enrichment of histone H3 acetylation within the *SNAI2* promoter upon Phorbol Ester treatment [142]. Histone acetylation promotes angiogenesis [143]. So far, VEGF signaling has been associated with angiogenesis. Transcription factor like FOXM1 binds in the forkhead response element (FHRE) region in the *VEGF* promoter which promotes H3 and H4 acetylation leading to VEGF induction [143]. FOXM1 has already been implicated in breast cancer. However, upon displacement of FOXM1 with FOXO3A in the promoter FHRE region, rapid deacetylation occurs due to the recruitment of HDAC2 [143]. As a result, VEGF expression is repressed.

Estrogen receptor plays an important role in breast carcinogenesis. Metastatic breast cancer cell line MDAMB-231 does not possess ER-α receptor and is a TNBC cell line while invasive MCF-7 is ER +ve [144]. Differential H3 and H4 acetylation within the ER-α promoter regulate ER-α expression in MDAMB-231 and MCF-7 cell lines [144]. pRB2/p130 molecular complex is recruited in ER-α promoter of MDAMB-231cells along with DNMT1. DNMT1 obstructs p300 (a HAT) from binding to the complex thereby maintaining a repression [144]. But in case of MCF-7, p300 binds with the complex in absence of DNMT1 and finally leads to expression of ER-α [145]. In case of HER-2 receptor, enrichment of acetylated H3 and H4 in the promoter leads to HER-2 over-expression [146]. HER-2 over-expression is found to have direct role in breast cancer metastasis [146].

In a study among 880 breast cancer patients, four different histone acetylation marks corresponded to distinct stage/fate of metastasis [135]. Lower expression of H3K9ac and H4K16ac were observed in lymph node stage samples [135]. H4K16ac is found to be positively associated with angiogenesis and is present at an early stage of cancer [147]. H3K18ac has been found to promote breast carcinogenesis [148]. H3K18ac causes transcriptional activation of gene promoters. Low H3K18ac level associated with high tumour grade [148]. Increased H3K4ac histone mark correlated to metastatic behavior of breast cancer [147]. H3K4 acetylation is also enriched within the promoters of EMT specific gene *VIMENTIN* in MDAMB-231 in comparison to MCF-7 and MCF-10A cells [149]. However acetylation in the promoters of epithelial markers like GATA3 and FOXA1in MCF-7 is more with respect to MDAMB-231 [149]. Apart from these, acetylated form of non-canonical variant H2A.Z is required for activation of p21 promoter in breast cancer cell line [150]. Another histone mark, H3K27ac has important role in breast cancer progression and is found to promote EMT [147, 150]. H3K27Ac is enriched at the tissue differentiation-inducing non-protein coding RNA (*TINCR*) non-coding RNA promoter that supports EMT in breast cancer [151].

#### C. Phosphorylation

Phosphate moiety is added to histone by kinases (writer) and removed by phosphatases (eraser) [152]. Phosphorylation is one of the widely studied mechanisms that has been implicated in cancer [153]. Phospho-histone variant ɣH2A.X has been already discussed under the histone variant section. ɣH2A.X is now routinely used as part of diagnosis in breast cancer [153]. Studies have shown that JMJD6earlier known for its demethylase and hydroxylase function has also intrinsic kinase activity. JMJD6 is found to cause phosphorylation of H2A.X histone variant at Tyr39 location [154]. Positive correlation between JMJD6 and H2A.X-phospho-Y39 has been observed in breast cancer cells [155]. Phospho-Y39-H2A.X causes upregulation of autophagy related genes like ATG1B, ATG5, ATG7 in triple negative breast cancer cell lines [155].

Significantly positive correlation between Oncotype DX recurrence and PhosphoH3 variants were observed among breast cancer patients [156]. In fact, the patients with higher phosphoH3 expression failed to survive due to metastasis during the trial period [156]. In biopsy sample of breast cancer patients, positive correlation was observed between phospho-histone H3 level and ki-67 proliferation index (n = 98) [157]. However, contradictory data showed that elevated phospho-histone has positive outcome amongst Asian TNBC patients [158].

Histone variant H3.3 has already been implicated in breast cancer. P21 (RAC1) activated kinase 1 (PAK1) is a kinase, which enters the nucleus upon mitotic induction where it causes phosphorylation of histone H3.3 at Ser10 residue [159]. H3.3 phosphorylation leads to condensation of chromosome and induction of mitosis leading to cell proliferation [159]. Another study has shown that how H3 phosphorylation is a part of signaling cascade that leads to breast cancer metastasis. Interestingly, IL-6 overexpression due to the genotoxic NF-κB activation, could also induce breast cancer metastasis [160]. Apart from these, phosphorylated H4 variant-H4S1ph and Phospho-histone H1 (pt146) have been implicated in breast cancer but their role in metastasis is yet to be confirmed [161, 162].

#### D. Ubiquitination

Protein ubiquitination is a dynamic process where there are two categories of players-ubiquitin enzyme (writer) and deubiquitin enzymes (eraser) [163]. Ubiquitination is possible on all the histone molecules and they have also been implicated in cancer [164]. So far most reported ubiquitin histone molecules are of H2A and H2B. Lower H2AK127ub1 and H2BK120ub1level associated with breast cancer [165–167]. USP22 (Ubiquitin specific protease 22) is an ubiquitin enzyme, which removes ubiquitin molecule from histone H2A, and H2B variants [168]. USP22 expression is induced in breast cancer and significantly associates with lymph node metastasis and ki-67 level [168]. However contrasting role of H2Bub is also reported [167]. In breast cancer of basal type, lower level of H2Bub is observed while in luminal breast cancer higher level of H2Bub is observed [167]. Thus, it is very important to diagnose properly theH2Bub level before application of ubiquitin mediated therapy.

#### E. PARylation

Unlike other histone post translational modifications, role of histone PARylation in breast cancer metastasis is scarcely reported. Like its other counterparts, PARylation also has writer (Poly ADP Ribose = PARP) and eraser (PolyADP Ribose glycohydrase = PARG) molecule [169]. PARP9 over-expression is significantly associated with breast cancer metastasis [170]. Upon knock-down of PARP9 by si-RNA, cell migrations were inhibited. PARP9 has been reported to enhance doxorubicin resistance via H4 ubiquitination and protecting the cell from further DNA damage [170]. EZH2 (Enhancer of Zeste 2), component of polycomb repressive complex (PRC2) is known for mediating breast cancer progression and metastasis via H3K27 tri-methylation [171]. PARylation of histone H3 decreases the affinity of EZH2 for histone H3 and as a result global reduction in H3K27 methylation is observed [171]. Further PARylation of EZH2 molecule itself also decreases the interaction of EZH2 with chromatin, leading to heterochromatin formation thereby reducing the chances of chromatin mediated interaction of different factors [171]. A summary of different histone modifications implicated in breast cancer metastasis has been represented in Table 3.

**Table 3:** Histone modifications in breast cancer metastasis

S. no.	Histone modification	Role in breast cancer metastasis	References
Methylation			
1.	H3K4	Oncogene JARID1A/1B interacts with pRb by demethylation of H3K4 Lower expression in basal carcinomas and Her-2 positive tumors Higher H3K4me3 is associated with breast tumor H3K4me3 is also regulated via PI3/AKT pathway in breast cancer H3K4me3 enrichment is found in the promoter of AURKB and E2F2 (AKT target) PRMT-5 causes H3K4 tri-methylation in the promoter of FOXP1	[124, 125, 135, 136]
2.	H3K27	Lower H3K27me3 is associated with breast cancer, tumor size, lymph node stage H3K27me3 is catalyzed by EZH2 which itself is an important player of EMT H3K27me3 is found in the promoter region of FOXC1, RAD51, CDH1, RUNX3, FOX-A1	[126–130]
3.	H3K79	DOT1L promotes H3K79 methylation Higher H3K79 methylation is observed in the BCAT1 promoter Causes genomic instability Promotes tumorigenesis	[131–134]
4.	H3R2	PRMT-5 causes H3R2 di-methylation in the promoter of FOXP1	[136]
5.	H4R3	Lower expression in basal carcinomas and Her-2 positive tumors Associated with lymph node stage H4R3me2 by PRMT1 causes tumorigenesis PRMT1 is recruited in the ZEB1 promoter for H4R3 di-methylation	[135, 136]
6.	H2A.Z.1.K101	H2A.Z.1.K101 undergoes di-methylation by SYMD3 in Cyclin A1 promoter region	[90]
Acetylation			
1.	H3	H3 acetylation in slug promoter causes EMT in phorbol ester treated MCF-7 PRMT7 causes lowering of H3 acetylation in cdh-1 promoter FOXM1 is recruited in the VEGF promoter where it causes H3 acetylation thereby promoting angiogenesis But when FOXO3a is enriched in VEGF promoter it recruits HDAC2 and causes deacetylation therefore repressing VEGF signal P300 (HAT) causes acetylation of H3 and H4 in ER-α promoter in MCF-7 and therefore active expression. While presence of DNMT in the promoter region of sER-α in MDAMB occludes p300 association thereby repressing it Association with HER-2 positive breast cancer. H3 and H4 acetylation is mainly found in the promoter region of Her-2 positive cells	[141–145]
2.	H3K4	Increased H3K4 acetylation mark is associated with metastatic behavior of sample H3K4 is enriched in the promoter region of VIM gene in MDAMB H3K4 mark is observed in the promoter region of GATA3 and FOXA1 in MCF-7 and MCF-10A	[147, 148]
3.	H3K9	Low H3K9 mark is associated with lymph stage sample	[136]
4.	H3K18	Associated with high grade tumour Enriched in the promoter of EMT specific genes	[147]
5.	H3K27	Promotes EMT	[147, 150]
6.	H4	PRMT7 causes lowering of H4 acetylation in cdh-1 promoter FOXM1 is recruited in the VEGF promoter where it causes H4 acetylation thereby promoting angiogenesis	[142, 143]
7.	H4K16	Low H4K16 mark is associated with lymph stage sample Positive association with angiogenesis and carcinogenesis	[135, 146]
8.	H2A.Z	Acetylated H2A.Z is required for p21 promoter activation in breast cancer cells	[149]
Phosphorylation			
1.	H2A.X	Phosphorylated H2A.X at ser139 i.e. ɣH2A.X is associated with breast cancer metastasis ɣH2A.X is recruited at DNA repair site upon DSBs H2A.X tyr39 also has positive correlation with breast cancer JMJD6 causes phosphorylation of H2A.X at tyr39 to activate autophagy related genes (ATG1B, ATG5 and ATG7) in TNBC	[57, 107]
2.	PhosphoH3	Positive correlation between oncotype genotyping test and PhosphoH3 is observed amongst breast cancer patients Possitive PhosphoH3 level is observed in breast cancer biopsy samples PhosphoH3 is found to be associated with positive outcome among Asian breast cancer patients PAK1 causes H3.3 phosphorylation which is implicated in breast cancer	[155–159]
3.	Phospho H1	Differential level of phospho histone H1 (pt146) is observed in different breast cancer cell lines	[162]
4.	Phospho H4	Phospho histone H4 (ser1) is found to be recruited at DSBs	[161]
Ubiquitination			
1.	H2Bub	Basal breast cancer has lower H2Bub level while luminal breast cancer has higher H2Bub level USP-22 causes deubiquitination of H2A and H2B histones. USP-22 has positive association with lymph node metastasis and ki-67 level	[164, 167, 168]
2.	H2AK119ub1	Low CRL4B expression leads to cell proliferation and invasion by causing global loss of H2AK119ub1	[166]
3.	H2AK127ub1	Lower H2AK127ub1 is observed in TNBC	[165]
4.	H2BK120ub1	Lower H2BK120ub1 is associated with breast cancer	[166]
PARylation			
1.	H3, H4	H3 PARylation decreases EZH2 affinity for H3 resulting in global loss in H3K27 methylation PARP9 over-expression is associated with breast cancer metastasis PARP9 enhances doxorubicin resistance via H4 ubiquitination	[170, 171]

The sole relevance of writing this review is to consolidate the different observations that has been generated in the context of histone epigenetics in breast cancer and thereby put forward the concept whether an amalgamation of these histone modifiers could be used to design any strategy that would help in the diagnosis of this disease. A substantial amount of information is already present wherein the histone modifiers along with other molecules or therapies have been successfully used for treating breast cancer. Basically, a significant understanding of histone epigenetics paved the way to exploit different histone modifiers as therapeutic targets in the treatment of breast cancer. In the next section, we will discuss on the different strategies undertaken so far.

### Exploiting histone epigenetics in designing strategies for breast cancer treatment

Being the most common cancer among women worldwide, the intensive research done on breast cancer made it possible for the patients to have a proper screening, early diagnosis and eventually receive treatment. Due to the heterogeneous nature of the cancer, the treatment modalities differ among individuals. Small molecule inhibitors of different signaling pathways, monoclonal antibodies, peptides and targeting histone modifiers or their functions have been studied in detail and have been successfully tested in clinical trials. The testing of these drugs and inhibitors first undergoes preclinical testing followed by clinical trials in different phases.

#### Preclinical models

Among the different factors studied in histone epigenetics, the role of HDACs has been significantly exploited to target breast cancer therapy. Preclinical studies in breast cancer cells have demonstrated the efficacy of different HDAC inhibitors (HDACi) including suberoylanilide hydroxamic acid (SAHA), trichostatin A (TSA), suberic bishydroxamate (SBHA), valproic acid (VPA). Use of SAHA (Vorinostat) in breast cancer cell lines MCF-7, MDAMB-231, MDAMB-435 and SKBR-3 induced growth inhibition and apoptosis [172]. TSA inhibit tumor growth of breast cancer cells by degradation of Cyclin D1 and inhibition of ER-α transcription in ER-α positive breast cancer cells [173]. SBHA led to the inhibition in proliferation and induction in apoptosis of MCF7 breast cancer cell line [174]. VPA demonstrated antiproliferative capacity in both HER-2 overexpressing and negative breast cancer cells and could induce apoptosis in TNBC cells as well [175, 176]. A long list of other HDACi like panobinostat, entinostat, sodium butyrate has also been tested in different types of breast cancer. A nice review focusing on recent use of HDAC inhibitors in preclinical studies have been well documented by Damaskos et al. [177]. In addition to HDACi, a significant number of small molecules targeting different signaling pathways have been tested in preclinical studies. Doxorubicin (DOX), a chemotherapeutic agent, has been effectively used in breast cancer malignancies but is associated with severe side-effects. A recent study showed that use of cholesterol depleting agent methyl-β-cyclodextrin (MCD) could reduce the effective dose of DOX but retaining its effect on loss in cell viability and inducing apoptosis in breast cancer cells [178]. Talazoparib, a Poly (ADP-ribose) polymerase (PARP) inhibitor, showed maximum efficacy in breast cancer cells due to its strong binding to DNA by trapping PARP–DNA complexes [179]. Presently, it is in phase III clinical trial. Patritumab, a monocloncal antibody, showed significant antitumor activity by inhibiting the formation of HER2/HER3 heterodimer [180]. Lapatinib, a dual reversible tyrosine kinase inhibitor for HER2 and EGFR receptors block the downstream ERK1/2 and PI3K/AKT signaling pathways thereby inducing the cell-mediated cytoxicity against breast cancer cells [181]. Preclinical studies in inflammatory breast cancer (IBC) cells showed that these cells survive the reactive oxygen species (ROS) associated death due to the presence of a signature oxidative stress response mechanism. But upon administration of Disulfiram, an FDA approved small molecule, supplemented with Cu, oxidative stress-mediated apoptosis was induced in multiple IBC cellular models [182]. Epoxyazadiradione, one kind of limonoids isolated from plant *Azadirachta indica*, could inhibit breast tumor growth by inhibiting PI3/AKT-dependent mitochondrial depolarization, restricting cell migration, angiogenesis while inducing apoptosis of TNBC and ER^+^ breast cancer cells [183]. In short, a considerable number of preclinical studies have been performed and equally a significant number is ongoing in different parts of the world. Concise reviews on the use of these models have been mentioned by Tong et al. and Wang and Xu [184, 185]. Although these models serve as the best option for studying and analyzing the role of different molecules in progression and treatment of breast cancer, however, neither of them could actually represent the humanized model of the disease. The pros and cons of using these models have been discussed in detail in the review by Holen et al. [186].

#### Clinical trials

Epigenetic modifiers including targeted therapies against histones and DNA methylation have undergone a substantial number of clinical trials for the treatment of different kinds of cancer. Presently, FDA has approved six epigenetic drugs for clinical application including azacitidine, 5-Aza-2′-deoxycytidine, SAHA (Vorinostat), romidepsin, belinostat, panobinostat and chidamide [187]. Clinical trials website (https://clinicaltrials.gov) showed 13 trials using either of these drugs alone in the treatment of breast cancer. These trials are in the category of completed or recruiting or active but not yet recruited. Additionally, these drugs in combination with other drugs are undergoing clinical trials as well. A search in clinicaltrial.gov with breast cancer and HDAC inhibitor showed 45 such trials including the same categories mentioned above.

1. Targeted therapies against histone methylation: Till date, clinical trials on breast cancer using targeted histone methylation therapy has not yet been reported as per our knowledge but quite a significant number of trials are either ongoing or completed in leukemia, lymphoma, endometrial cancer, prostrate cancer, melanoma among others. A list of all these undergoing trials has been mentioned in the recently published review by Cheng et al. [187].
2. Targeted therapies against histone acetylation: A considerable number of trials against histone acetylation has been reported in breast cancer as well as in other cancers mentioned in https://clinicaltrials.gov website. Currently 14 such trials have been undertaken for breast cancer patients. Among these trials, few have completed, some are recruiting while few of them are active but not yet recruiting [187]. The drugs belong to anti-HDAC, sirtuins inhibitors or BRD (BET) (bromodomain) inhibitors. Sirtuins are a class of HDACs while bromodomains and extra-terminal domain (BET) are epigenetic readers that could bind acetylated histone at the regulatory regions of DNA including promoter, enhancer and thereby regulate transcription. So, inhibitors of these molecules would disrupt the mechanisms associated with histone acetylation at different loci.
3. Histone modification and DNA methylation: The major components of epigenetics comprise histone modifications, DNA methylation, microRNA and non-coding RNAs. Although this review involve histone epigenetics in breast cancer but the status and regulation of DNA methylation also dictate the initiation or inhibition of breast cancer and hence is equally important in the proper understanding of breast cancer and its metastasis. The concerted role of histone modifications and DNA methylation becomes quite obvious while considering the treatment strategies that have been undertaken by various groups whether in preclinical or clinical studies. Mechanistic studies in breast cancer cell lines proved the beneficial existence of these two modifications in regulation of breast cancer metastasis. Histone lysine demethylase KDM2A could inhibit TET2, the DNA demthylase enzyme, and promote DNA methylation and silencing of tumor suppressor genes in TNBC cells [188]. Loss of an environmentally induced gene called *MDIG* (mineral dust-induced gene) induced both DNA methylation and H3K9me3 resulting in increased metastasis of aggressive breast cancer cells [189]. Direct physical interaction between histone H3K9 trimethylase SETDB1 and DNA methyl transferase enzyme DNMT3A resulted in promoting silencing of cancer cell specific genes studied in TNBC MDAMB-231 cells [190]. Similar crosstalk has been proven in cancers of other origins as well. This closely knit crosstalk have been further exploited to design strategies that could enhance the efficiency of breast cancer treatment. In case of advanced breast cancer, a trial to introduce the combination of Azacitidine and entinostat drugs has been initiated (#NCT01349959) wherein Azacitidine is a cytidine analog whereas entinostat is an HDAC inhibitor [187].
4. Combination therapy.

Present day anticancer treatment involves cytotoxic chemotherapy, targeted therapy, radiotherapy or immunotherapy. These anticancer agents are however cytotoxic in nature and with time the cancer cells develop resistance towards these agents. Also, these agents cannot distinguish between the normal and cancer cells. So, a combined therapy of using anticancer drug along with other chemotherapeutic agent would introduce the administration of a lower dosage of anticancer agent thereby facilitating the efficacy and the enhanced antitumor effect of the treatment. Histone epigenetic modifiers especially the HDAC inhibitors are being successfully used as part of this combination therapy.

a. Chemotherapy: HDAC inhibitors in addition to other chemotherapeutic drugs have been shown to offer better respite in cancers of different origin [191]. A phase I/II clinical trial is underway with the combination of olaparib and vorinostat in patients with refractory lymphomas (NCT03259503). A phase II clinical trial of Vorinostat combined with tamoxifen has been used for the treatment of patients with hormone therapy-resistant breast cancer that demonstrated an increase in DNA damage, growth inhibition and cell death [192]. Clinical trial on use of HDAC inhibitor along with cell cycle checkpoint inhibitor showed effective anti-tumor activity in metastatic ER^+^ breast cancer cells [193]. Another clinical phase III trial (NCT02115282) is active but not yet recruiting for treating patients with recurrent hormone receptor-positive breast cancer that is locally advanced or metastatic by the combined use of the drugs entinostat, exemestane, goserelin, goserelin acetate. A search in clinical trials.gov showed 30 such trials either in the category of completed, recruiting or active but not recruiting status using HDAC inhibitors along with other drugs in breast cancer patients.

b. Radiotherapy: One of the common and early form of treatment of cancer is using radiotherapy that causes cell death due the DNA double strand breaks. But, with time the cells even become resistance to this with alleviated level of DNA repair mechanism of the body. Experimental evidences showed the role of HDACi in DNA damage and repair signaling and hence this avenue is being exploited in combination to radiotherapy. Preclinical study in lung carcinoma cells showed that HDAC inhibitor SAHA could sensitize these cells to radiation with minimum effects to normal cells that would enhance the effect of radiotherapy of the cancer cells [194]. Another preclinical study in breast cancer cells MCF7, proved that a proper balance between HDACs and HATs is necessary to maintain the histone acetylation thereby the compaction of the chromatin to resist the development of resistant of cancer cells to radiation therapy [195]. So, an early diagnosis of tumor HDAC activity would increase the efficiency of HDAC/radiotherapy strategy of treating cancer cell [196]. Clinical trials.gov website showed only one such completed trials on brain metastases although not specifically arising from breast cancer metastasis (NCT00838929). A combination therapy of vorinostat along with radiation was used for the treatment of patients with brain metastases. Interestingly, similar combination therapy has been approached in more numbers in cancers of other origins. Presently, 15 such trials are in different stages of clinical trials including completed, recruiting or active but not recruiting status. Hence, it is obvious from the number of these trials that the combination therapy of histone epigenetics and radiation is indeed a valid option that is being seriously considered to give a better alternative to the patients suffering from cancer.

## Discussion

In this review, we have attempted to present a comprehensive study on the role of various histone-mediated processes in the context of breast cancer metastasis. With the current knowledge we can have an idea about the histone landscape of a breast cancer metastatic model. These might open newer avenue for therapeutic interventions. Metastatic breast cancer is one of the most aggressive cancer conditions. Apart from chemotherapeutic treatment no other alternative could offer an efficient strategy for the treatment of metastatic breast cancer. Targeting histone-mediated pathways for drug delivery might reduce the burden of the unbearable side effects of the present therapies. We also got an idea about developing a new set of epigenetic biomarkers that could be exploited for identifying the progression of diseased condition.

As breast cancer arises from heterogenic condition, it might not be feasible for a single biomarker to determine the metastasis condition for all categories or subtypes of breast cancer. Each of the breast cancer subtypes have their distinct mechanism of pathogenesis. Biomarker for one subtype might not serve for another, therefore leading to false diagnosis. However, instead of a single biomarker, a panel of these epigenetic regulators can be screened in order to have an idea about the probability of developing breast cancer metastasis in patients. Here we have included five epigenetic factors namely APLF, HJURP, MacroH2A.1, ɣH2AX and H2Bub1, whose association has been found with different sub-types of metastatic breast cancer (Table 4).

**Table 4:** Probable list of epigenetic biomarkers targeting all subtypes of breast cancer

S. no.	Biomarkers	Association with hormonal receptors	Breast cancer subtypes
1.	APLF	TNBC	Basal
2.	HJURP	ER−ve, PR−ve	Luminal A
3.	MacroH2A.1	TNBC HER-2	Basal (more), Luminal (less) HER-2-enriched
4.	ɣH2AX	TNBC HER2−ve HER2+ve	Basal, HER2 enriched Luminal A/B (less) Luminal B (less than TNBC)
5.	H2Bub1	ER+ve TNBC	Luminal A/B (more expression) Basal (less expression)

Rationale for choosing APLF and HJURP amongst all other histone chaperones is that, APLF over-expression is associated with TNBC/basal type breast cancer metastasis while HJURP over-expression is associated with luminalA breast cancer metastasis. So by including these, we can screen for both basal and luminalA subtype breast cancer samples. Just like HJURP, increase H2Bub1 expression is also associated with luminal cancer, while significantly reduced H2Bub1 expression is present in basal type. As MacroH2A interact with HER2, it could be an important identifier in case of HER2+ve breast cancer. ɣH2AX has been implicated as a marker in prognosis of breast cancer of triple negative and HER2−/HER2+ subtypes [197]. Additionally, ɣH2AXis associated with DNA repair mechanism and hence could demonstrate the overall stability of a genome after chemotherapy. As breast cancer could relapse even after chemotherapy sessions, so the ɣH2AX level in those patients could act as an indicator of how stable is the genome after the therapy and hence could be included in the biomarker panel.

This review has tried to fill the gap of creating a histone epigenetic landscape for metastatic breast cancer. One important aspect that is still not clear is whether epigenetic modifications are consequences of aberrations in epigenetic modifiers or they part of the cancer etiology? The hallmarks of cancer basically indicated cancer as a disease of the genome [198]. But, the same hallmarks could be achieved only by change in epigenome as well [198] and thereby inflicts a question mark on cancer being a disease of the genome. The genetic mutations in chromatin modifiers or remodelers can lead to deregulation of epigenome thereby contributing to cancer associated epigenetic aberrations. The gain-of-function EZH2 mutations present in several lymphomas, is responsible for aberrant histone H3K27me3 resulting the blockage of B-cell development [199]. While on the other hand, change in epigenome might be completely due to non-genetic deregulation. If we consider the case of solid tumor like breast cancer, a cohort of 70 primary TNBC samples analyzed by TCGA displayed unique epigenetic status in different breast cancer subtype. H3K9ac mark associated with HER2-positive and TNBC tumors [200] whereas H3K27me3 marks were significantly reduced in luminal-B, HER2-positive, and TNBC tumors, but was increased in the luminal-A subtype [201]. This sort of data could be invariably used for the development of clinical options and also to study therapy resistance towards drugs. Now, whether the methylation happened post the onset of cancer or the particular methylation gave rise to cancer is yet be deciphered. The knowledge on how epigenetic events lead to tumour progression and metastasis is very limited at present. Our molecular tools are yet to achieve that precision level to understand the difference between cause and result of cancer. Hence current studies and the information availed from the existing literature is not at all conclusive. A substantial portion of the picture is still missing. Contradictory results, significant work on cell lines rather than in vivo also contribute to non-specific results as the epigenetic factors function in association with other factors and not individually.

Moreover, significant mechanistic details are yet to be revealed to understand what causes the primary breast tumors to become metastatic in nature. Extensive sequencing studies proved that mutation is not the causal effect of changing from primary to metastatic. A primary tumor cell has to perform several functions to metastasize to a distant organ and that include detachment from the tumor/site, invasion into the stroma and then circulatory system, migration, penetration and anchorage to the new organ site, modulation of the surrounding microenvironment for its growth and survival and at last forming a new tumor at the new site. All these steps involve a close association with the local environment of the tumor cells and hence stand a high chance of undergoing epigenetic modifications within the loci of the cells. The progression of breast tumor cells is associated with EMT and CDH1 is one of the fundamental genes in inhibition of metastasis. During metastasis, a significant loss in expression of CDH1 has been observed mostly due to the hypermethylation corresponding to both DNA methylation and H3K27 trimethylation [202]. More than the genetic mutations, it’s the epigenetic modifications that act as drivers in change of fate of primary tumor cell to become metastatic. DNA methylation is well conserved between primary and corresponding metastatic tumours in prostate cancer [203]. However, intratumoural DNA methylation heterogeneity correlated to genomic copy number patterns and metastatic progression of prostrate cancer [204]. On the other hand, DNA methylation especially outside CpG rich region mark metastasis associated methylation of genes in breast cancer, which indicate a difference in methylation in primary tumor vs. metastatic breast cancer [205]. The entire picture is yet to be revealed, what we have only is the trailer right now. So, there is a need to study the role of remaining HCs, HVs and their modifications in metastatic breast cancer. Metastatic breast cancer is a multifactorial and a complex disease condition, lot of heterogeneity [heterogeneity because of different molecular sub-types]. In order to assess the predictability of a biomarker, it is better to test on patients belonging to same subtypes. In that way we will have a better chance of understanding altered molecular mechanism that a cell undergoes during metastasis. Quite understandably, the same biomarker might play different role via different pathways in different breast cancer subtypes. Sample heterogeneity could not reveal any conclusive mechanism and thereby cannot assure a particular biomarker that can foresee the appearance of metastatic breast cancer. So, to avoid this heterogeneity, five epigenetic factors could be screened as biomarkers that might be exploited to detect the probability of developing metastasis of breast cancer.

## Conclusion

A single biomarker could not detect or predict the metastatic condition for all categories or subtypes of breast cancer. This in-depth review on the study of different epigenetic modifiers in the context of breast cancer metastasis led to the formulation of a panel of epigenetic factors namely APLF, HJURP, MacroH2A.1, ɣH2AX and H2Bub1, that could be exploited to detect the potential of breast cancer to become metastatic in nature.

## Data Availability

Not applicable.

## Abbreviations

4EBP1: Eukaryotic translation initiation factor 4e binding protein 1
AD: Acidic domain
ANP32: Acidic leucine rich Nuclear Phosphoprotein 32
AKAP8: Protein A-Kinase Anchor Protein 8
APLF: Aprataxin PNK-like factor
ARID1A: AT-rich interaction domain 1A
ASF1: Anti-silencing factor 1
ATG1B/ULK2: unc-51 like autophagy activating kinase 2
ATG5: Autophagy related 5
ATG7: Autophagy related 7
ATM: Ataxia Telangiectasia Mutated
ATRX: Alpha thalassemia/mental retardation syndrome X-linked
AURKB: Aurora kinase B
BCAT1: Branched chain amino acid transaminase 1
BET: Bromodomains and extra-terminal domain
BRD: Bromodomain
CAF1: Chromatin assembly factor 1
CDH1: Cadherin 1 [E-cadherin]
CDK8: Cyclin dependent kinase 8
CENP: Centromere protein A
CBD: CENP-A binding domain
ChIP: Chromatin immunoprecipitation
c-Met: MET proto-oncogene
C-Myc: MYC proto-oncogene
DAXX: Death domain associated protein
Ddx17: DEAD-Box helicase 17
Ddx5: DEAD-Box helicase 5
DEK: DEK proto-oncogene
DNMT: DNA methyl transfease
DOT1L: DOT1 like histone lysine methyltransferase
DOX: Doxorubicin
DRE: DEK response element
DSC2: Desmocollin 2
E2F1: E2F transcription factor 1
E2F2: E2F transcription factor 2
ECM: Extra cellular matrix
EGFR: Epidermal growith factor receptor
EMT: Epithelial-to-mesenchymal transition
ER: Estrogen receptor
ER-α: Estrogen receptor alpha
ErbB2: Erb-B2 receptor tyrosine kinase 2
ERK1/2: Extracellular signal regulated kinase ½
EZH2: Enhancer of zeste 2 polycomb repressive complex 2 subunit
FACT: Facilitates chromatin transcription
FERMT1: Fermitin family member 1
FHRE: Forkhead response element
FOXA1: Forkhead Box A1
FOXC1: Forkhead Box C1
FOXP1: Forkhead Box P1
GSC: Goosecoid Homeobox
H2AFY: MacroH2A.1 histone
HAT: Histone acetyl transferase
HBD: Histone binding domain
HC: Histone chaperone
HDAC: Histone deacetylase
HER: Human epidermal growth factor
HIF1α: Hypoxia inducible factor 1 subunit alpha
HIRA: Histone cell cycle regulator A
HIST1H2BJ/E: Histone cluster 1 H2B family member J/E
HJURP: Holiday junction recognition protein
HM: Histone modification
HP1α: Heterochromatin protein 1α
HV: Histone variant
IDC: Invasive ductal carcinoma
IHC: Immunohistochemistry
ITGB8: Integrin subunit beta 8
JARID1A: Lysine demethylase 5A
JMJD6: Jumonji domain containing 6
KDM5A: Lysine demethylase 5A
LOX: Lysyl oxidase
LTED: Long term estrogen-deprived
MCD: Methyl-β-cyclodextrin
MMP3: Matrix metallopeptidase 3
MMP9: Matrix metallopeptidase 9
MMTV: Mouse mammary tumor virus
MRN: MRE11-RAD50-NBN
MSH4: MutS protein homolog 4–5
MSH5: MutS protein homolog 5
mTOR: Mammalian target of rapamycin
NAP1: Nucleosome assembly protein 1
NFkB: Nuclear factor kappa B subunit 1
NPM1: Nucleophosmin
Pak-1: P21 [RAC1] activated kinase 1
PARP: Poly[ADP-Ribose] polymerase 1
PCNA: Proliferating cell nuclear antigen
PDCD4: Programmed cell death 4
P13K: Phosphatidylinositol-3-kinase
PR: Progesterone receptor
PRC2: Polycomb Repressor Complex 2
PRMT1: Protein arginine methyltransferase 1
PRMT5: Protein arginine methyltransferase 5
PRMT7: Protein arginine methyltransferase 7
PTEN: Phosphatase and tensin homolog
PTM: Post-translational modifications
RAD51: RAD51 recombinase
RB: Retinoblastoma
RON: Recepteur d’Origine Nantais
ROS: Reactive oxygen species
RUNX3: Runt-related transcription factor 3
SAHA: Suberoylanilide hydroxamic acid
SAM: *S*-adenosylmethionine
SBHA: Suberic bishydroxamate
SCF: Skp Cullin F-box
SETDB1: SET domain bifurcated histone lysine methyltransferase 1
SIM: SUMO-interacting Motif
SKP2: S-phase kinase associated protein 2
SMYD3: SET and MYND domain containing 3
SNAI1: Snail Family Transcriptional Repressor 1
SNAI2: Snail Family Transcriptional Repressor 2
SPT16: Suppressor of Ty 16 homolog
SSRP1: Structure specific recognition protein 1
STAT3: Signal transducer and activator of transcription 3
TCF3: Transcription factor 3
TCGA: The Cancer Genome Atlas
TFCP2L1: Transcription factor CP2 like 1
TGFB: Transforming growth factor beta
TFF1: Trefoil factor 1
TINCR: Tissue differentiation-inducing non-protein coding RNA
TNBC: Triple negative breast cancer
TRAF6: TNF receptor associated factor 6
TSA: Trichostatin A
TWIST1: Twist basic helix-loop-helix transcription factor 1
UGT8: UDP glycosyltransferase 8
USP22: Ubiquitin specific peptidase 22
VPA: Valproic acid
VEGF: Vascular endothelial growth factor
VIM: Vimentin
YY1: YY1 transcription factor
ZEB1: Zinc Finger E-Box Binding Homeobox 1
ZID: H2A.Z Interacting domain
ZNF367: Zinc finger protein 367
